# Lipid Transfer Protein Allergens: A Comprehensive Overview of Current Knowledge

**DOI:** 10.3390/ijms27052132

**Published:** 2026-02-25

**Authors:** Magdalena Rydzyńska, Tomasz Rosada, Bernadetta Kosztulska, Magdalena Grześk-Kaczyńska, Natalia Ukleja-Sokołowska

**Affiliations:** Department and Clinic of Allergology, Clinical Immunology and Internal Diseases, Ludwik Rydygier Collegium Medicum in Bydgoszcz, Nicolaus Copernicus University in Toruń, 87-100 Toruń, Poland; medtom@op.pl (T.R.); bernadetta-kosztulska@wp.pl (B.K.); magdalenagrzesk@gmail.com (M.G.-K.); uklejan@cm.umk.pl (N.U.-S.)

**Keywords:** allergy, food allergy, allergen, non-specific lipid transfer protein, Pru p 3

## Abstract

Non-specific lipid transfer proteins (nsLTPs) constitute a widely distributed family of plant allergens with substantial clinical relevance, particularly in food allergy. Their marked thermal and proteolytic stability enables them to provoke reactions ranging from mild local symptoms to severe anaphylaxis. This narrative review synthesises current knowledge on nsLTP allergens, focusing on their molecular characteristics, taxonomic distribution, exposure routes, and clinical impact. Major allergenic sources include fruits, nuts and seeds, vegetables and cereals, as well as various pollens. Across these sources, Pru p 3 has emerged as the central and most extensively studied allergen, frequently acting as the primary sensitiser and exhibiting broad cross-reactivity with homologous nsLTPs from diverse plant species. Despite growing evidence, significant knowledge gaps remain regarding sensitisation pathways, environmental modifiers, and phenotype stratification. Continued research is required to improve diagnostic precision and guide the development of targeted therapeutic strategies for patients with nsLTP-mediated allergy.

## 1. Introduction

Knowledge about environmental allergens is continually expanding, and the list of known molecules is updated each year with newly identified proteins.

This also applies to non-specific lipid transfer proteins (nsLTPs), which are important pan-allergens from the perspective of clinical practice.

They were first described in plants more than 40 years ago [1,2,3]. These proteins are present in fruits, vegetables, grains, and plant pollen, and their presence is associated with a substantial risk of anaphylactic reactions following food exposure. The first identified allergen in this group was isolated from peach (*Prunus persica*) more than 25 years ago, and its characterisation became a reference point for the diagnosis of LTP allergy [4].

In 2025, the list of nsLTPs was expanded to include additional newly discovered proteins, and, according to the WHO/IUIS Allergen Nomenclature Sub-Committee, by early 2026, 60 allergens belonging to this family had already been registered [5]. This highlights the importance of these widespread proteins, which remain incompletely understood.

The aim of this review is to systematise the current state of knowledge regarding non-specific lipid transfer proteins and to present their clinical relevance.

## 2. Non-Specific Lipid Transfer Proteins

Lipid transfer proteins are small, non-glycosylated plant-derived proteins belonging to the prolamin superfamily. Their molecular weight ranges from 6 to 10 kDa. Based on molecular size and polypeptide chain length, two main classes of these proteins are distinguished: LTP1, with a molecular weight of 9–10 kDa and a length of approximately 90 amino acids, and LTP2, with a molecular weight of 6–7 kDa and an average length of approximately 70 amino acids.

LTPs play a key role in lipid transport, particularly during the formation of plant surface structures. They are also involved in defence mechanisms against pathogens, regulation of growth, and cellular signalling processes. A characteristic structural feature of these proteins is the presence of cysteine residues forming stabilising disulphide bonds, which confer exceptional resistance to physical and chemical factors [3,6]. This resistance includes high temperatures and digestive enzymes, enabling LTPs to induce allergic reactions even after thermal processing of food.

LTPs are widely distributed in the plant kingdom. They occur in fruits, vegetables, grains, nuts, tree and weed pollen, as well as in latex. The highest concentrations are observed in the skin and outer layers of fruits and vegetables and in seeds [3,7]. It should be emphasised that the LTP content of individual plant products is variable and depends on species, degree of ripeness, and storage conditions [8].

Available reports on this group of proteins suggest the existence of additional differences between LTP1 and LTP2. At the same time, there is a lack of analyses focused on the direct comparison of these two groups, particularly with regard to their clinical relevance.

Most of the available data concern type 1 proteins. This group includes, among others, the major peach allergen Pru p 3, as well as LTPs present in commonly consumed fruits such as apples, cherries, and grapes. These proteins are described as a frequent cause of severe allergic reactions, including anaphylaxis, and their localisation within fruits is reported mainly in the skin and outer layers of the flesh [3].

In contrast, among the LTP allergens currently listed by the WHO, only four belong to type 2 proteins. All of them are food allergens and occur in peanuts and walnuts, celery root, and tomato seeds. Literature data regarding anaphylaxis following their consumption are limited. Case reports and cohort analyses predominantly concern the more frequently occurring fruit allergens from the Rosaceae family. There are insufficient data to conclude that sensitisation to LTP1 is associated with a higher risk of severe anaphylactic reactions compared with LTP2.

Due to the small number of identified representatives of this group, it is difficult to unequivocally assess whether the localisation of LTP2 and the clinical manifestations of the allergic reactions they induce differ from those observed for LTP1.

Knowledge of LTPs remains insufficient. The number of identified allergens is limited despite their widespread occurrence, and many remain undiscovered. Consequently, it is currently not possible to clearly differentiate allergic syndromes associated with LTP1 and LTP2. A more comprehensive assessment of the clinical significance of differences between these groups requires further molecular and clinical studies. Table 1 summarises the key features of type 1 and type 2 nsLTPs.

## 3. Allergy to LTP—Clinical Presentation

The clinical presentation of LTP allergy is characterised by substantial variability. Symptoms may range from mild manifestations, often underestimated by patients, to life-threatening reactions. Mild manifestations include urticaria, including contact urticaria, angioedema, and oral allergy syndrome. More severe presentations include full-blown anaphylaxis and, in extreme cases, anaphylactic shock [9,10].

In some patients, peeled fruits and vegetables may be well tolerated and do not elicit allergic symptoms. Nevertheless, even in such cases, the risk of allergic reactions is not completely eliminated, particularly in the presence of cofactors. Among the most important cofactors that exacerbate the clinical course of LTP allergy is physical exercise [10].

Other cofactors implicated in allergic reactions include the use of non-steroidal anti-inflammatory drugs, alcohol consumption, concomitant infections, exposure to stressful situations, and menstruation [11].

Current descriptions of the mechanisms by which physical exercise acts as a cofactor in allergic reactions focus on increased splanchnic blood flow and damage to the intestinal epithelium resulting from anaerobic metabolism. These changes lead to increased intestinal permeability and enhanced allergen bioavailability [12,13]. The potential role of exercise-induced changes in plasma osmolality, which may trigger basophil activation, has also been highlighted [14,15].

Another important group of cofactors comprises non-steroidal anti-inflammatory drugs. By inhibiting prostaglandin synthesis, these agents impair the protective function of the gastrointestinal mucosa, thereby promoting mucosal damage and increasing intestinal permeability [16]. Reports also suggest the possibility of direct mast cell activation, as well as the contribution of elevated adenosine levels to the amplification of allergic reactions [15].

Alcohol may also act as a cofactor by disrupting tight junctions in the intestinal epithelium, thereby compromising intestinal barrier integrity [17,18]. In addition, its metabolite, acetaldehyde, has been shown to enhance mast cell degranulation, which may further promote the development of allergic reactions [19].

The phenomenon referred to as LTP syndrome describes a multisystem, often clinically severe, cross-reactive allergy to multiple plant-derived sources, resulting from sensitisation to stable and widely distributed non-specific lipid transfer proteins [20].

## 4. Epidemiology

Allergy to non-specific lipid transfer proteins most commonly occurs in inhabitants of Mediterranean countries. However, in recent years, a clear increase in the number of reported cases has been observed in regions of Central Europe, highlighting the growing clinical relevance of this type of allergy [3,21]. The majority of reports concern the major peach allergen, Pru p 3.

An analysis of 23,000 patients with a history of food allergy, allergic rhinitis, asthma, atopic dermatitis, urticaria, or anaphylaxis, who underwent ISAC microarray testing in Italy, revealed sensitisation to Pru p 3 in 9.79% of cases. In this group, sensitisation to another LTP, the inhalant allergen Parietaria judaica allergen Par j 2, was observed significantly more frequently; specific IgE to Par j 2 was detected in 25.71% of patients [22]. When interpreting these results, it should be noted that they refer to sensitisation and not necessarily to clinically manifest allergy.

Another Italian study demonstrated that among 351 patients with fruit and vegetable allergy confirmed by specific IgE testing and clinical history, LTPs were responsible in as many as 60% of cases. Among the 210 patients with LTP allergy, allergic reactions were most frequently triggered by peach (n = 178) and apple (n = 74). At the same time, it was shown that fruit and vegetable allergy was significantly more common in southern Italy than in central or northern regions [23].

In a further Italian study evaluating patients with anaphylaxis, LTPs were identified as the causative factor in approximately 30% of cases [24]. Likewise, in a study investigating individuals with food-induced anaphylaxis associated with physical exertion, Pru p 3 was identified as the causative allergen in 78% of the 82 patients examined [25].

These findings underscore the high risk associated with LTP allergy, particularly given that in some patients allergic symptoms occur exclusively in the presence of cofactors. This highlights the significant role of both the allergen itself and external factors in the development of severe clinical reactions.

Numerous case reports of LTP allergy have also been published from other regions of Europe. However, detailed epidemiological data remain limited, and available studies generally involve relatively small patient cohorts [9,26].

Although LTP allergy has been classically associated with the Mediterranean area, particularly Spain and Italy, where Pru p 3 represents the dominant primary sensitiser and systemic reactions are common, increasing evidence indicates a broader and heterogeneous geographic distribution [9,10]. In Central Europe, inhalant-driven primary sensitisation, especially via mugwort-derived Art v 3, appears more relevant and is frequently associated with concomitant pollen allergy [27]. Northern Europe has traditionally been dominated by PR-10-related pollen–food syndrome; however, clinically relevant LTP sensitisation, although less prevalent than in Southern Europe, is increasingly recognised [28]. Emerging data from Asia and the Americas suggest that LTP-mediated reactions are not confined to Europe, although epidemiological evidence remains fragmented and likely underestimates true prevalence [10,29].

Despite the substantial clinical impact of LTP sensitisation in Mediterranean populations, most available evidence derives from single-country studies, predominantly conducted in Italy, which may limit generalisability. Many analyses are based on selected cohorts referred for specialised testing rather than population-based samples, potentially leading to overestimation of prevalence. Moreover, reliance on molecular sensitisation profiles without systematic confirmation by oral food challenges complicates differentiation between clinically relevant allergy and asymptomatic sensitisation. Larger, multicentre, population-based studies are therefore needed to more precisely define the true epidemiological burden, geographical variability, and predictors of severe phenotypes.

## 5. LTP Sources

Sources of LTPs include fruits, vegetables, cereals, and plant pollen. Current knowledge regarding most of these proteins remains limited, and research aimed at elucidating their structure, homology, and clinical relevance is ongoing. A compilation of the LTPs identified to date is presented in Table 2.

## 6. LTP Characteristic

Although proteins from the nsLTPs family exhibit high sequence and structural homology, considerable differences are observed within this group, including variations in sensitisation mechanisms and the clinical manifestations of allergic reactions. To organise the available data, these proteins have been classified according to the predominant route of exposure, distinguishing LTPs associated with alimentary, inhalant, and contact exposure.

A concise overview of the best-characterised members of this protein family is presented below. Despite significant advances in research, the available data for some LTPs remain very limited and often consist solely of their identification. For this reason, the present review focuses on proteins for which the scope of available information is most extensive.

### 6.1. Food Allergens

*Act c 10/Act d 10*

Gold kiwifruit (*Actinidia chinensis*) and green kiwifruit (*Actinidia deliciosa*) are sources of type 1 LTPs with a molecular mass of approximately 10 kDa. Their amino acid sequences show 96–97% identity between the two cultivars [30].

In a study investigating the prevalence of sensitisation to individual kiwifruit components, Act d 10 emerged as the most frequent trigger of symptoms among individuals residing in Southern Europe, accounting for 22% of cases. The analysis drew on data from the EuroPrevall cohort and included 311 patients [31].

Structural homology has also been demonstrated between Act d 10 and Ara h 9 (56%) as well as between Act d 10 and Pru p 3 (47%) [32].

*Api g 2*

Celery (*Apium graveolens*) is a source of both type 1 and type 2 lipid transfer proteins [5,33].

Api g 2, belonging to the nsLTP1 family and with a molecular weight of 9 kDa, is localised predominantly in celery stalks. Sequence homology of Api g 2 with other LTPs ranges from 32% to 67%, with the highest similarity observed with LTPs derived from saltbush and clove. Its thermostability and resistance to pH changes have been confirmed [34].

The clinical phenotype of Api g 2-dependent allergy is heterogeneous. In an analysis of 26 Italian patients sensitised to other LTPs, specific IgE to Api g 2 was detected in 15 individuals; within this subgroup, five patients avoided celery due to severe reactions, five presented with mild mucocutaneous symptoms, and five remained asymptomatic [34]. Similarly, in a cohort of 786 Italian individuals evaluated using the ISAC microarray test, 25.6% showed sensitisation to Api g 2. Isolated reactivity to Api g 2 was observed in 32 subjects; among these, consumption of celery stalks elicited clinical symptoms in 10 patients [35].

*Api g 6*

The type 2 LTP derived from *Apium graveolens*, present in the celery root, is a protein with a molecular weight of approximately 7 kDa and was first described in 2013. Similar to other LTPs, it exhibits high thermal stability and resistance to digestive processes [36].

In a study involving 32 Austrian patients, specific IgE to Api g 6 was detected in 37.5% (12 individuals). Notably, Api g 6 shows a high degree of sequence homology—71%—with the LTP from the European olive, whereas its similarity to Api g 2 is only 21% [36].

*Ara h 9*

The peanut (*Arachis hypogaea*) is a source of three LTP family proteins identified to date. The best-characterised of these is Ara h 9, an nsLTP1 with a molecular mass of approximately 9.8 kDa. Two isoforms of the recombinant protein have been described: rAra h 9.0101 and rAra h 9.0201. Their amino acid sequences share 62–68% homology with Pru p 3 [37].

Numerous studies have examined patients with clinically confirmed peanut allergy, analysing their sensitisation profiles to individual allergen components. In a study involving 117 children with confirmed peanut allergy, specific IgE to Ara h 9 was detected in 62% of participants [38]. Comparable results were obtained in another Italian study evaluating sensitisation patterns in 48 children with peanut allergy, in which 58% tested positive for Ara h 9 [39]. A further analysis of sera from 42 patients with peanut allergy identified specific IgE to Ara h 9 in 19 individuals [40].

In a study examining 115 patients from the United States, Spain, and Sweden, Ara h 9 emerged as the most frequently recognised peanut allergen exclusively in the Spanish population, being responsible for allergy in more than 60% of patients. In contrast, the prevalence was only 7.7% in the American cohort and 14.3% in the Swedish cohort. Moreover, 60% of Spanish patients who tested positive for Ara h 9 were monosensitised [41].

In a large Italian multicentre study involving 395 children and adults with confirmed peanut allergy—of whom 35% experienced local reactions, 38.2% systemic reactions, and 26.6% anaphylaxis—77% were found to have specific IgE to Ara h 9 [42].

Collectively, these findings clearly demonstrate that sensitisation to Ara h 9 is strongly influenced by geographical latitude. In Mediterranean populations, Ara h 9 represents the dominant peanut allergen component responsible for sensitisation.

*Aspa o 1*

Asparagus (*Asparagus officinalis*) is the source of Aspa o 1, an allergen identified in 2002 as an nsLTP1 with a molecular mass of approximately 9 kDa. It is, to date, the only described allergen from asparagus. Its identification was made possible through the analysis of 18 patients who developed respiratory symptoms, anaphylaxis and/or contact urticaria following ingestion of, or exposure to, asparagus, and who additionally exhibited positive skin prick test responses and specific IgE to asparagus extract. Skin test reactivity to the isolated asparagus LTP was positive in 50% of these patients [43].

*Cas s 8*

Chestnut *(Castanea sativa*) is a source of a type 1 nsLTP, with exposure to this allergen occurring predominantly through the alimentary route [5,44].

*Cit l 3/Cit r 3/Cit s 3*

Lemon (*Citrus limon*), mandarin (*Citrus reticulata*), and orange (*Citrus sinensis*) are citrus fruits that contain type 1 nsLTPs with a molecular mass of approximately 9 kDa. Purified allergens from orange (nCit s 3) and lemon (nCit l 3) display very high similarity in their N-terminal amino acid sequences, with 18 out of 20 residues being identical [45].

In a study involving 27 patients with oral allergy syndrome symptoms triggered by orange consumption, specific IgE to the purified allergens was detected in 54% of patients for nCit l 3, 48% for nCit s 3, 46% for rCit s 3, and 37% for rPru p 3. Cross-reactivity between citrus LTPs and peach LTPs was also demonstrated [45].

*Cor a 8*

A type 1 LTP with a molecular mass of 9 kDa derived from *Corylus avellana* was first described in 2002 [46]. Its high thermostability and resistance to acidic pH have been confirmed [47].

It has been demonstrated that Cor a 8-related allergy is associated with an increased risk of severe anaphylactic reactions. Furthermore, monosensitisation to Cor a 8, compared with polysensitisation to Cor a 8 and Cor a 1, has been linked to a more severe clinical course of allergic reactions [48].

In a Chinese study using serum inhibition assays, Cor a 8 was shown to be inhibited by Art v 3, confirming cross-reactivity between these proteins and suggesting the possibility of primary sensitisation via the inhalant route. However, it should be emphasised that the study population predominantly exhibited symptoms of oral allergy syndrome [49].

In a large analysis of 800 Italian children assessed between 2010 and 2020, the overall prevalence of sensitisation to Cor a 8 was found to be 30%. The authors also reported a significant increase in sensitisation rates over the decade, with the prevalence in 2020 being 36 percentage points higher than in 2010, underscoring the growing clinical relevance of Cor a 8 in the paediatric population [50].

*Jug r 3*

Jug r 3 is a type 1 LTP with a molecular mass of 9 kDa derived from *Juglans regia* (walnut). Its identification was made possible through the analysis of 46 patients who reported symptoms following walnut consumption; in this group, 72.8% were found to have specific IgE to Jug r 3. Furthermore, complete inhibition of the protein by Pru p 3 has been demonstrated, supporting the hypothesis that sensitisation is secondary and most likely results from cross-reactivity with peach [51].

*Lac s 1*

*Lactuca sativa* (lettuce) belongs to the Asteraceae family. In this species, a protein of approximately 9 kDa has been identified and classified as a type 1 nsLTP, recognised as the major lettuce allergen [52,53].

In a group of 14 patients presenting with allergic symptoms after lettuce consumption, the isolated LTP was shown to elicit clinical reactions in 9 individuals. Its identification was further confirmed using an inhibition assay with peach and cherry extracts [52]. In another study involving 29 patients with lettuce allergy, with or without concomitant peach allergy, two isoforms of Lac s 1 were identified, whose amino acid sequences displayed 66% identity with Pru p 3. Moreover, 72% of the patients experienced anaphylaxis after consuming lettuce [53].

*Mal d 3*

Apples (*Malus domestica*) constitute an important source of type 1 nsLTPs with a molecular mass of 9 kDa. The thermostable properties of this protein have been unequivocally confirmed [54].

Researchers from the United Kingdom evaluated LTP concentrations in 53 apple cultivars grown in Italy and 35 cultivars originating from the Netherlands, demonstrating that LTP levels may vary by up to a hundred-fold between different varieties [55]. In another study analysing ten apple cultivars (Golden, Golden Perlim, Reineta, Reineta Parda, Fuji, Verdedoncella, Granny Smith, Pink Lady, Royal Gala, Starking), the highest LTP concentration was observed in the Starking cultivar [56]. Further analyses have shown that LTP content in apples depends not only on the cultivar but also on the degree of ripeness and storage time—more mature fruits contain higher amounts of LTP, whereas prolonged storage results in a gradual decline in LTP levels [8].

In an analysis of 81 patients with confirmed apple allergy conducted by Gómez et al., the clinical sensitisation profiles of symptomatic individuals were assessed. Among the study population, 35 patients presented with oral allergy syndrome, whereas 46 experienced systemic reactions. Mal d 3 was identified as the causative allergen in 37% of the entire cohort, with only a 0.5-percentage-point difference in prevalence between patients with local versus systemic symptoms, suggesting a comparable role of Mal d 3 in both clinical phenotypes [57].

*Pru av 3*

*Prunus avium* (sweet cherry) belongs to the Rosaceae family and contains a type 1 nsLTP with a molecular mass of approximately 10 kDa. The resistance of Pru av 3 to enzymatic digestion and high temperatures has been experimentally confirmed [58].

The LTP from sweet cherry exhibits a high degree of amino acid sequence identity with LTP allergens from peach (Pru p 3–88%), apricot (Pru ar 3–86%), and maize (Zea m 14–59%) [59].

An analysis of six sweet cherry cultivars revealed no significant differences in LTP concentrations among them. However, chemical peeling and syruping were shown to markedly reduce LTP content, confirming that this allergen is primarily localised in the peel [60].

In a German study including a total of 143 patients from Central Europe and Spain with cherry allergy, marked geographical differences in sensitisation to rPru av 3 were identified. Specific IgE to rPru av 3 was detected in 20 of 22 Spanish patients (91%), whereas positive results were observed in only 13 of 121 patients (11%) from Central Europe [61].

*Pru p 3*

*Prunus persica* (peach) represents a major source of nsLTPs, with Pru p 3 recognised as the key allergen responsible for primary sensitisation to LTPs. This protein is a type 1 nsLTP with a molecular mass of approximately 10 kDa and consists of 91 amino acid residues [4]. It remains the best-characterised LTP allergen identified to date.

Pru p 3 has been shown to be located predominantly in the peach peel [62]. In a Spanish study involving 57 children with positive specific IgE to peach, 96% of patients exhibited IgE reactivity to Pru p 3, while more than 90% tolerated the fruit once the peel had been removed [63]. The primary nature of sensitisation to Pru p 3 has been repeatedly confirmed in inhibition assays, which demonstrated suppression of IgE reactivity to other LTPs by Pru p 3, indicating its initiating role in the development of LTP syndrome [51,52].

Interestingly, peach LTP has been reported to induce not only food-related allergic symptoms but also contact urticaria following skin exposure to fresh fruit [64].

Attempts at sublingual and oral immunotherapy with peach extract or fresh peach juice have yielded promising results. However, the available studies are limited by small sample sizes, ranging from single-patient case reports to the largest cohort of 56 participants. Reported efficacy rates—defined as an increased tolerated dose of peach—range from 72% to 100% of study participants [65]. In one study, six months of sublingual immunotherapy in 33 patients resulted in a three- to nine-fold increase in the tolerated peach dose [66]. Another study involving 32 patients who completed the protocol demonstrated improved tolerance not only to peach but also to peanut, further supporting the concept of Pru p 3 as a primary sensitizer and a central driver of cross-reactivity within the LTP family [67].

Unfortunately, long-term outcome data are scarce; most follow-up periods range from one to three years, and the persistence of tolerance beyond this interval remains uncertain [65]. Moreover, the available studies employed heterogeneous immunotherapy protocols, differing in dosing regimens, treatment duration, and outcome definitions, which limits direct comparison of results and precludes firm conclusions regarding efficacy.

In recent years, emerging therapeutic strategies for food allergy have increasingly incorporated biologics. Omalizumab has been shown to reduce reaction severity and to facilitate food reintroduction or desensitisation. Preliminary evidence also suggests potential benefits of dupilumab or ligelizumab, although data remain very limited and controlled studies are needed [68]. While no dedicated trials of biologics in LTP allergy currently exist, a recently published case report described successful continuation of LTP sublingual immunotherapy facilitated by dupilumab administration [69].

Collectively, these findings indicate that immunotherapy targeting Pru p 3 is promising, and biologics may serve as supportive agents in selected high-risk individuals. Nevertheless, robust prospective studies are required before standardised therapeutic protocols can be established.

*Sola l 3/Sola l 6/Sola l 7*

The tomato (*Solanum lycopersicum*) is a source of LTPs, with three distinct LTPs identified in its fruit: two belonging to the type 1 nsLTP family and one to the type 2 nsLTP family. Their molecular masses range from 7 to 12.5 kDa. Importantly, these proteins display differential localisation within the fruit [5,70].

Sola l 3, initially referred to as Lyc e 3, is a type 1 nsLTP identified in tomato peel. In a study involving 35 Spanish patients with documented allergic reactions after tomato consumption, specific IgE to Sola l 3 was detected in 29% of participants. Moreover, attempts have been undertaken to genetically modify tomatoes to reduce expression of this protein, resulting in a substantial—10- to 100-fold—decrease in histamine release by human basophils stimulated with extracts from transgenic fruits compared with control extracts [70]. These findings suggest the potential for the development of hypoallergenic tomato cultivars.

Sola l 6 is a type 2 nsLTP with a molecular mass of 7 kDa and, unlike Sola l 3, is localised not in the peel but in the seeds. This protein consists of 68 amino acids and exhibits 41% sequence identity with Pru p 3 [71].

Sola l 7, also exclusively localised in the seeds, is a type 1 nsLTP with a molecular mass of 12.5 kDa.

An analysis involving 22 patients with allergic reactions of varying severity from mild oral allergy syndrome to full-blown anaphylaxis underscored the clinical relevance of seed-derived tomato LTPs. In this cohort, positive skin prick test results were observed in 95.5% of patients for fresh tomato, 68% for seed extract, and 50% for the purified seed nsLTP. Furthermore, 71.4% of patients with anaphylaxis demonstrated positive immunoblotting results for Sola l 7. The amino acid sequence identity between Sola l 6 and Sola l 7 was only 32%. Sola l 7 showed the greatest similarity to other allergenic LTPs: Act d 10 (53%), Pru p 3 (41%), and Ara h 9 (51%) [72]. Evaluation of digestive stability revealed that although all three tomato LTPs are resistant to enzymatic hydrolysis, Sola l 7 displays the highest resistance to digestion [73].

*Vit v 1*

*Vitis vinifera* is the source of the allergen Vit v 1, a 9 kDa protein present in both fresh grapes and wine [74].

Its identification was made possible through the analysis of 14 patients reporting allergic reactions after consuming grapes or wine. In 10 of the 14 patients (71%), specific IgE binding to the 9 kDa Vit v 1 protein was demonstrated in immunoblots of grape extract. Cross-reactivity between Vit v 1 and Pru p 3 was also confirmed, showing that IgE binding to the grape LTP was completely abolished following pre-incubation with 5 µg of peach LTP [74].

In another study involving 37 patients with acute allergic reactions after grape consumption, all participants were found to be sensitised to Vit v 1 [75]. Additional reports in the literature describe further cases of full-blown anaphylaxis triggered by the ingestion of grapes or wine [76,77].

Researchers in Germany conducted an oral immunotherapy trial aimed at inducing tolerance to grapes, wine, and raisins in a single patient. A three-day initiation protocol enabled the achievement of a maintenance dose equivalent to 20 g of grapes. After two months, tolerance to 66.5 mL of white wine was confirmed during a controlled food challenge [77]. This case illustrates the therapeutic potential of immunotherapy in LTP-mediated food allergy.

*Zea m 14*

Maize (*Zea mays*) is a source of a type 1 nsLTP with a molecular mass of approximately 9 kDa. This protein was identified as the major maize allergen based on an analysis of sera from 22 patients who experienced systemic reactions after consuming maize. In 19 of the 22 patients (86%), the sera demonstrated binding to a 9 kDa protein. Determination of its N-terminal sequence confirmed that it is a lipid transfer protein belonging to the nsLTP family [78].

### 6.2. Inhalant Allergens

*Amb a 6*

*Ambrosia artemisiifolia*, ragweed, is the source of two isoforms of the allergen Amb a 6, a type 1 nsLTP with a molecular mass of 10 kDa [79].

An analysis of sera from 150 patients sensitised to ragweed demonstrated that approximately 30% possessed specific IgE to Amb a 6. In 47 cases, sensitisation was confirmed using ELISA, while in 45 cases it was verified with the ImmunoCAP system. In six patients, IgE levels specific to Amb a 6 exceeded those directed against the major allergen Amb a 1, underscoring the potential clinical relevance of this protein. Furthermore, homology analysis revealed that Amb a 6 shares the highest amino acid sequence identity—51%—with the LTP of sunflower, whereas identity with other LTPs was below 40% [80].

*Art v 3*

*Artemisia vulgaris* (mugwort), a perennial plant of the Asteraceae family, is an important source of type 1 nsLTPs with a molecular mass of 12 kDa. Art v 3, the major LTP allergen of mugwort, is predominantly localised in the pollen wall [81].

Numerous reports indicate that Art v 3 may function as a primary inhalant sensitiser, subsequently leading to the development of the LTP syndrome. In some patients with IgE reactivity to both Art v 3 and Pru p 3, Art v 3 behaves as the initiating sensitising allergen [82].

In a study by Zhong-Shan Gao et al., 70 patients were evaluated: 31 sensitised exclusively to mugwort, 24 sensitised to both mugwort and peach, and 15 sensitised exclusively to peach. The authors demonstrated that among Chinese patients with peach allergy, the primary sensitising allergen was not peach but Art v 3 from mugwort pollen. These conclusions were supported by both sIgE measurements and inhibition assays [83].

In another cohort of 50 patients with both inhalant and food allergy, Art v 3 was the most frequently detected LTP component, with the highest mean sIgE levels, suggesting its role as a precursor of LTP sensitisation in this population [84]. Furthermore, in a study of 240 Chinese patients with clinically confirmed mugwort allergy and positive sIgE to mugwort pollen, the prevalence of sensitisation to Art v 3 was 53%, further underscoring its significance in the pathogenesis of mugwort allergy [85].

Researchers performing nasal provocation tests showed that monosensitisation to Art v 3, without concomitant sensitisation to food-derived LTPs, may lead to symptoms of allergic rhinitis [86].

*Can s 3*

*Cannabis sativa* (hemp) is used both in the textile industry and as a source of psychoactive substances. It has been shown to contain a type 1 nsLTP with a molecular mass of 9 kDa [87]. In recent years, a considerable global increase in the medical and recreational use of Cannabis sativa has been observed [88].

The relevance of LTPs in cannabis allergy is supported by a study involving 120 patients reporting symptoms after contact with Cannabis sativa. Episodes of anaphylaxis were recorded in 25 individuals, and in 72% of these cases, Can s 3 was identified as the eliciting factor. Moreover, patients sensitised to Can s 3 frequently exhibited co-sensitisation to other LTPs, indicating the involvement of Can s 3 in a broader cross-reactivity network. Allergic reactions in this group were more often associated with cofactors, highlighting the increased susceptibility of patients sensitised to cannabis-derived LTPs to more severe clinical manifestations in their presence [89].

Early suggestions of primary sensitisation to Cannabis sativa leading to secondary food allergy to LTP-containing foods arose from case reports. A 28-year-old man who experienced upper airway symptoms (sneezing, rhinorrhoea), eyelid oedema, and ocular pruritus after smoking marijuana subsequently developed anaphylaxis after consuming tomato and pepper. The same patient also experienced contact urticaria after touching peach peel, anaphylaxis after eating figs, and oral allergy syndrome in response to apples, almonds, aubergines, and chestnuts. During diagnostic evaluation, Can s 3 was identified as the allergen responsible for these reactions [90,91].

These observations led to the recognition of Can s 3 as the major allergen of Cannabis sativa. In the context of LTP allergy, this allergen is notable because, despite its classification as an inhalant allergen, exposure to Cannabis sativa may occur via inhalation, ingestion, or skin contact. Inhalant exposure may result from pollen, occupational exposure during cultivation, and both active and passive smoking [88]. Clinical reports corroborate these exposure routes, documenting cases of anaphylaxis following ingestion of hemp flour-containing cake or contact with hemp leaves [92]. Anaphylactic shock after consuming hemp seeds has also been described [93].

These findings indicate a broad spectrum of allergic risks associated with the use of hemp, encompassing inhalant-, contact-, and food-related reactions, all of which may result in secondary sensitisation to other potentially hazardous LTP allergens. This phenomenon is of particular concern given the increasing global availability of cannabis and the expanding diversity of exposure routes.

*Pla a 3*

London plane tree (*Platanus acerifolia*) is a source of the inhalant allergen Pla a 3, a type 1 nsLTP with a molecular mass of approximately 10 kDa. This allergen has been shown to share 58.3% amino acid sequence identity with the peach allergen Pru p 3 [94].

Interesting findings have emerged from studies evaluating the effects of nitrogen dioxide (NO_2_) and ozone (O_3_), two major components of air pollution, on the properties of plane tree pollen. It has been demonstrated that NO_2_ and O_3_ can damage the pollen cell membrane, leading to increased release of Pla a 3 into the atmosphere. Moreover, exposure of pollen to these pollutants induces structural modifications in Pla a 3, enhancing its immunogenicity and potentially intensifying the allergic response [95].

*Tri a 14*

Wheat (*Triticum aestivum*) is the source of the inhalant allergen Tri a 14, a type 1 nsLTP with a molecular mass of 9 kDa. Its thermostability and resistance to enzymatic digestion have been experimentally confirmed [96].

The clinical relevance of Tri a 14 has been particularly highlighted in the pathogenesis of baker’s asthma, a form of occupational asthma caused by chronic inhalational exposure to wheat flour. In a Spanish study involving 37 patients with baker’s asthma, 95% demonstrated sensitisation to Tri a 14 in skin prick testing. Additionally, a bronchial provocation test with Tri a 14 was performed in 27 patients, yielding positive results in 82% [97]. In another study including 40 patients with this form of occupational asthma, specific IgE to Tri a 14 was detected in the sera of 60% of affected individuals. Notably, none of the patients reported symptoms following ingestion of wheat-containing foods, suggesting that the sensitisation pathway for Tri a 14 is predominantly inhalational rather than alimentary [98].

Conversely, another case-based analysis of patients with wheat allergy demonstrated that Tri a 14 may also act as a causative factor in systemic allergic reactions [99].

These findings underscore the complex clinical nature of Tri a 14-related allergy, encompassing both inhalant sensitisation and a potential risk of severe post-exposure reactions. In light of current evidence, further research is required to better characterise the associated clinical phenotypes and the sensitisation mechanisms linked to this protein.

### 6.3. Contact Allergen

*Hev b 12*

Hev b 12 is a lipid transfer protein derived from *Hevea brasiliensis* (the Brazilian rubber tree) and is currently the only LTP described with contact allergenic properties. Latex, the milky sap produced by this plant, serves as the primary raw material in the rubber industry. To date, sensitization to Hev b 12 has not been implicated in the pathogenesis of latex–fruit syndrome, suggesting that this protein exhibits a clinical profile distinct from the classical latex allergens associated with this condition [100].

## 7. LTP Allergy Profile

The list of LTP family allergens identified to date comprises 60 proteins. However, only a limited subset of these allergens is available in routine commercial diagnostics. Both singleplex and multiplex diagnostic approaches allow for the detection of specific IgE antibodies to only a small number of the known LTP allergens.

Among multiparametric methods, the ALEX2 Explorer Allergen test (Macroarray Diagnostics, Vienna, Austria) enables the measurement of specific IgE antibodies to the following LTP allergen components: nAct d 10, rAra h 9, rArt v 3, rApi g 2, rApi g 6, rCan s 3, rCor a 8, rJug r 3, rMal d 3, rPar j 2, rPla a 3, Pru p 3, nSola l 6, rTri a 14, nVit v 1 and rZea m 14. Its extended version, ALEX3, additionally allows for the detection of specific IgE to Fra a 3, Hel a 3, Len c 3, Pis s 3, Pru av 3, and rOle e 7 [101].

Another widely used semi-quantitative multiparametric test is ImmunoCAP ISAC (Thermo Fisher Scientific, Waltham, MA, USA), which includes the following LTP components: rAra h 9, nArt v 3, rCor a 8, rJug r 3, rOle e 7, rPla a 3, rPru p 3, rPar j 2 and rTri a 14 [102].

The FABER test (Centri Associati di Allergologia Molecolare, CAAM, Rome, Italy), which has since been withdrawn from the market, additionally enabled the detection of specific IgE antibodies to Gly m 1 and Pun g 1 [103].

Overall, the use of these diagnostic methods allows for the assessment of 24 of the currently known LTP family allergens.

In singleplex diagnostics using the ImmunoCAP system, key nsLTP components are available, including Pru p 3 (peach), Mal d 3 (apple), Cor a 8 (hazelnut), Jug r 3 (walnut), Ara h 9 (peanut), Tri a 14 (wheat), nOle e 7 (olive), rPar j 2 (Parietaria judaica), nArt v 3 (mugwort), and Can s 3 (hemp), offered as individual allergen components [102]. Additionally, Allerg-O-Liq (Dr. Fooke Laboratorien, Neuss, Germany) catalogues indicated the possibility of measuring sIgE to rFra a 3, while Immulite (Siemens Healthcare Diagnostics, Forchheim, Germany) offers the detection of sIgE to rPru av 3 [20,104,105].

These data highlight significant diagnostic and research limitations in the comprehensive assessment of sensitisation to LTP family allergens. Despite the growing number of identified LTP allergens, their availability in routine diagnostic practice remains limited.

The clinical profile of patients with LTP allergy is highly heterogeneous, and the primary source of sensitisation is often difficult to determine unequivocally. This is supported by case reports describing sensitisation induced by less commonly considered allergen sources [49,82,83,91]. Such variability significantly complicates the clinical assessment of patients with suspected LTP syndrome.

Pru p 3, the peach allergen, remains the most widely used marker in the diagnosis of LTP allergy. Previous studies have identified it as the most common cause of primary sensitisation within this protein family, while the high structural similarity among individual LTP allergens promotes extensive cross-reactivity [21]. For this reason, diagnostic strategies based on the measurement of specific IgE antibodies to Pru p 3 appear justified as a first step in the diagnostic work-up [20].

At the same time, studies by Morales et al., employing Pru p 3 inhibition assays with a range of fruits containing LTP allergens, failed to demonstrate inhibition for many of them, indicating limitations of Pru p 3 as a universal marker of sensitisation to all LTP allergens [106].

Although Pru p 3 may represent a useful diagnostic tool in cases with an unclear triggering factor, numerous reports emphasise its insufficient role in the comprehensive assessment of the sensitisation profile [20,107].

Moreover, available data indicate that Pru p 3 is not always the primary sensitising allergen. Cases have been described in which patients with clinically relevant LTP allergy showed no detectable sIgE to Pru p 3 but demonstrated sensitisation to other components of this allergen family [20]. Some studies have further suggested that the breadth of the sensitisation profile may correlate with the risk of more severe clinical reactions; in one Italian cohort, anaphylaxis occurred more frequently in patients sensitised to five or more LTP components [108,109]. However, these observations have not been consistently confirmed across all analyses.

The development of molecular diagnostics enables the assessment of broader sensitisation profiles through the simultaneous measurement of multiple allergen components. This approach allows for a more precise characterisation of sensitisation mechanisms and potential sources of primary sensitisation. At the same time, multicomponent diagnostics raise important clinical concerns, as they carry the risk of detecting sensitisation without clear clinical relevance, potentially leading to overinterpretation of results and complicating therapeutic decision-making.

In light of the available evidence, the diagnosis of LTP allergy remains challenging. Although Pru p 3 serves as the most commonly used marker of primary sensitisation, it does not always reflect the full sensitisation profile of the patient. Expanded LTP profiling using multicomponent assays allows for a more detailed assessment of sensitisation mechanisms and potential primary allergen sources but may also reveal clinically irrelevant sensitisation.

Thus, the question remains whether the determination of a broad LTP panel truly increases diagnostic value or whether, in most cases, marker-based assessment using Pru p 3 is sufficient. A definitive answer requires further studies that take into account both clinical outcomes and the risk of severe allergic reactions.

**Scheme 1:**
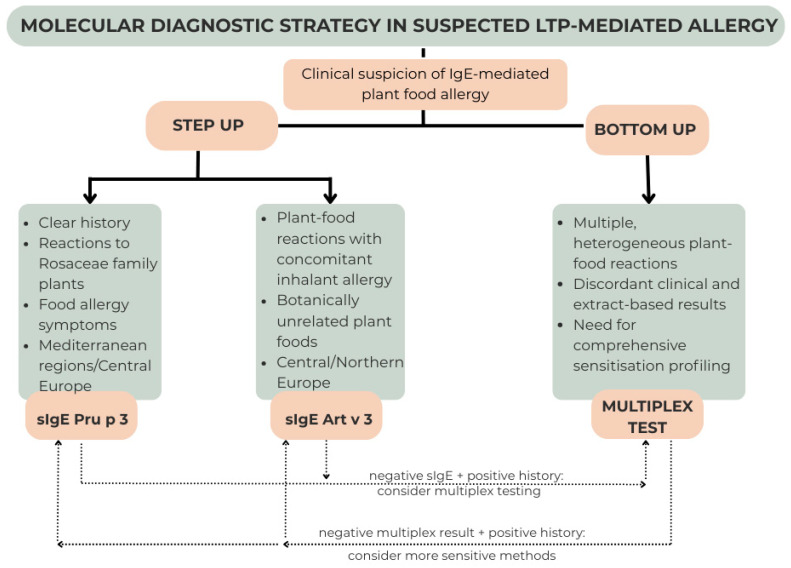
Molecular diagnostic strategy in suspected LTP allergy.

It should be emphasised that much of the current evidence regarding LTP sensitization profiles derives from retrospective analyses or highly selected cohorts referred to tertiary allergy centres, frequently in Mediterranean countries. Sample sizes in many studies remain limited, and prospective validation in broader, population-based settings is lacking. Furthermore, correlations between the extent of molecular sensitization and clinical severity have not been consistently confirmed across independent cohorts, and in many cases rely on sensitization patterns without systematic confirmation by standardised oral food challenges. These limitations complicate the interpretation of diagnostic profiling and underscore the need for carefully designed prospective studies integrating molecular data with well-characterised clinical phenotypes. Scheme 1 illustrates the proposed molecular diagnostic algorithm.

The cornerstone of food allergy diagnosis remains a carefully obtained clinical history. Skin prick testing with commercial extracts or native prick-by-prick testing should be performed as first-line investigations, as they are simple, accessible, and cost-effective tools that confirm IgE-mediated sensitisation in the appropriate clinical context [110].

Extract-based testing may be sufficient to establish the diagnosis of peach allergy in patients with a clear history of immediate reactions and concordant test results. However, positivity to whole extracts does not identify the underlying allergenic protein family. Since peach contains multiple relevant allergens, including LTP (Pru p 3), PR-10 (Pru p 1), and profilin (Pru p 4), extract positivity alone does not allow differentiation between LTP-mediated allergy and other pollen–food syndromes.

Therefore, molecular diagnostics is required to confirm LTP-mediated allergy and to establish the diagnosis of LTP syndrome. Identification of specific LTP components (e.g., Pru p 3 or other plant-derived LTPs) enables accurate phenotypic characterisation, improved risk stratification, and differentiation from typically milder PR-10- or profilin-driven reactions.

At the same time, indiscriminate use of broad molecular panels increases the likelihood of detecting asymptomatic sensitisation without clinical relevance. Such findings should not be overinterpreted, as sensitisation in the absence of symptoms does not warrant therapeutic intervention.

Given the still incomplete understanding of primary sensitisers, regional sensitisation patterns, and their association with clinical severity, diagnostic tools should be applied in a rational and clinically driven manner. In line with EAACI recommendations, molecular testing should complement—but not replace—a thorough history and appropriately selected first-line investigations [110].

## 8. Future Perspectives

Despite significant advances in understanding LTP-mediated allergy, several important questions remain. Improved characterisation of primary sensitisation pathways and their geographical variability is needed to better predict clinical phenotypes and to enable risk stratification, particularly for severe reactions and cofactor-dependent presentations.

Further refinement of diagnostic strategies remains a priority. The clinical utility of component-resolved diagnostics requires validation across diverse populations, especially in regions where the primary sensitising sources may differ. Establishing unified diagnostic algorithms that integrate clinical history, skin testing, molecular profiling, and standardised oral food-challenge protocols would improve diagnostic accuracy and help distinguish clinically relevant allergy from asymptomatic sensitisation. In addition, a clearer definition of molecular patterns associated with severe phenotypes could support personalised risk assessment and prevention strategies.

Finally, standardisation and optimisation of immunotherapeutic approaches, supported by larger multicentre studies with harmonised clinical endpoints and adequate long-term follow-up, are essential to clarify the durability of benefit and safety. The potential role of biologics as adjunctive therapy also warrants rigorous, controlled evaluation.

## 9. Summary

Non-specific lipid transfer proteins constitute a widely distributed family of plant allergens with significant clinical relevance in both food and inhalant allergy. Their pronounced thermal stability and resistance to digestion make them a particularly hazardous group of proteins, capable of eliciting reactions that span a broad clinical spectrum—from mild symptoms to severe systemic responses. Although several LTP allergens have been thoroughly characterised, current knowledge of the family as a whole remains incomplete. The available evidence highlights the broad taxonomic distribution of LTPs and their substantial potential for cross-reactivity; however, important knowledge gaps persist. Further research is warranted to better elucidate the molecular properties, sensitisation pathways, and clinical phenotypes associated with these proteins.

## Figures and Tables

**Table 1:** Summary of key features of type 1 and type 2 nsLTP.

Feature	LTP1	LTP2	Comment/Level of Certainty
Molecular weight/length	~9–10 kDa; ~90 aa	~6–7 kDa; ~70 aa	Well-established structural distinction
Thermal & proteolytic stability	High	High	No evidence of clinically relevant differences
Number of allergens identified	55 allergens (widely distributed)	4 allergens (peanut, walnut, celery root, tomato seeds)	Major imbalance in available data
Plant sources	Fruits (Rosaceae), nuts, cereals, vegetables, pollens, latex	Primarily seeds (peanut, walnut, celery root, tomato)	LTP2 distribution remains poorly characterised
Tissue localisation	Often in fruit peel, outer layers, seeds	Mainly seeds/storage tissues	Too few LTP2 representatives for firm conclusions
Clinical relevance	Frequent systemic reactions; anaphylaxis reported (e.g., Pru p 3)	Limited clinical data; severity unclear	Current evidence insufficient to compare risk
Primary sensitiser potential	Well documented (Pru p 3, Art v 3)	Unknown	No data supporting LTP2 as a primary sensitiser
Cross-reactivity	Extensive intra-family cross-reactivity	Presumed but minimally studied	Most data extrapolated from LTP1 studies

**Table 2:** Currently described nsLTPs according to allergen.org [5].

	Allergen	Source	Type	Molecular Mass	Route of Exposure
1.	Act c 10	Gold kiwi fruit	nsLTP1	10 kDa	Oral
2.	Act d 10	Green kiwi fruit	nsLTP1	10 kDa	Oral
3.	Amb a 6	Short ragweed	nsLTP1	10 kDa	Inhalant
4.	Api g 2	Celery	nsLTP1	9 kDa	Oral
5.	Api g 6	Celery	nsLTP2	7 kDa	Oral
6.	Ara h 9	Peanut, groundnut	nsLTP1	9.8 kDa	Oral
7.	Ara h 16	Peanut, groundnut	nsLTP2	8.5 kDa	Oral
8.	Ara h 17	Peanut, groundnut	nsLTP1	11 kDa	Oral
9.	Art an 3	Sweet wormwood	nsLTP1	10 kDa	Inhalant
10.	Art ar 3	Silvery wormwood	nsLTP1	10 kDa	Inhalant
11.	Art ca 3	Wormwood	nsLTP1	10 kDa	Inhalant
12.	Art d 3	Desert wormwood	nsLTP1	10 kDa	Inhalant
13.	Art gm 3	Russian wormwood	nsLTP1	10 kDa	Inhalant
14.	Art la 3	Mugwort	nsLTP1	10 kDa	Inhalant
15.	Art si 3	Sieversian wormwood	nsLTP1	10 kDa	Inhalant
16.	Art v 3	Mugwort, wormwood	nsLTP1	12 kDa	Inhalant
17.	Aspa o 1	Asparagus	nsLTP1	9 kDa	Oral
18.	Bra o 3	Cabbage	nsLTP1	9 kDa	Oral
19.	Bro p 3	Paper mulberry	nsLTP1	12–15 kDa	Inhalant
20.	Can s 3	Indian hemp	nsLTP1	9 kDa	Inhalant
21.	Cas s 8	Chestnut	nsLTP1	9 kDa (red) 12–13 kDa (non-red)	Oral
22.	Cit l 3	Lemon	nsLTP1	9.6 kDa	Oral
23.	Cit r 3	Tangerine	nsLTP1	9 kDa	Oral
24.	Cit s 3	Sweet orange	nsLTP1	9.46 kDa	Oral
25.	Cor a 8	Hazelnut	nsLTP1	9 kDa	Oral
26.	Fra a 3	Strawberry	nsLTP1	9 kDa	Oral
27.	Hel a 3	Sunflower	nsLTP1	9 kDa	Oral
28.	Hev b 12	Para rubber tree	nsLTP1	9 kDa	Contact
29.	Jug r 3	English walnut	nsLTP1	9 kDa	Oral
30.	Jug r 8	English walnut	nsLTP2	9 kDa	Oral
31.	Lac s 1	Cultivated lettuce	nsLTP1	9 kDa	Oral
32.	Len c 3	Lentil	nsLTP1	9 kDa	Oral
33.	Lup an 3	Narrow-leaved blue lupin	nsLTP1	11 kDa	Oral
34.	Mal d 3	Apple	nsLTP1	9 kDa	Oral
35.	Mor n 3	Black mulberry	nsLTP1	10 kDa	Oral
36.	Mus a 3	Banana	nsLTP1	9 kDa	Oral
37.	Ole e 7	Olive	putative- LTP	9.5 kDa	Inhalant
38.	Par j 1	Pellitory of the wall	nsLTP1	15 kDa	Inhalant
39.	Par j 2	Pellitory of the wall	nsLTP1	10–14 kDa	Inhalant
40.	Par o 1	Pellitory	nsLTP1	15 kDa	Inhalant
41.	Pha v 3	Green bean	nsLTP1	8.8–9.0 kDa	Oral
42.	Pis s 3	Pea	nsLTP1	9.5 kDa	Oral
43.	Pla a 3	London plane tree	nsLTP1	10 kDa	Inhalant
44.	Pla or 3	Oriental plane tree	nsLTP1	11 kDa	Inhalant
45.	Pru ar 3	Apricot	nsLTP1	9 kDa	Oral
46.	Pru av 3	Sweet cherry	nsLTP1	10 kDa	Oral
47.	Pru d 3	European plum	nsLTP1	9 kDa	Oral
48.	Pru du 3	Almond	nsLTP1	9 kDa	Oral
49.	Pru p 3	Peach	nsLTP1	10 kDa	Oral
50.	Pun g 1	Pomegranate	nsLTP1	9 kDa	Oral
51.	Pyr c 3	Pear	nsLTP1	9 kDa	Oral
52.	Rub i 3	Red raspberry	nsLTP1	11 kDa	Oral
53.	Sin a 3	Yellow mustard	nsLTP1	12.3 kDa	Oral
54.	Sola l 3	Tomato	nsLTP1	9 kDa	Oral
55.	Sola l 6	Tomato	nsLTP2	7 kDa	Oral
56.	Sola l 7	Tomato	nsLTP1	12.5 kDa	Oral
57.	Tri a 14	Wheat	nsLTP1	9 kDa	Inhalant
58.	Tri tu 14	Durum wheat	nsLTP1	9.2 kDa	Oral
59.	Vit v 1	Grape	nsLTP1	9 kDa	Oral
60.	Zea m 14	Maize	nsLTP1	9 kDa	Oral

## Data Availability

No new data were created or analyzed in this study. Data sharing is not applicable to this article.
